# Exploring the Global Legacy of Professor Calayampudi Radhakrishna Rao: A Review of His Life and Biostatistical Contributions

**DOI:** 10.7759/cureus.71504

**Published:** 2024-10-15

**Authors:** Naga Rajeev L, S Johnson, Helan Rajan, Pratap B Kaushik

**Affiliations:** 1 Community Medicine, Dr. D. Y. Patil Medical College, Hospital and Research Centre, Dr. D. Y. Patil Vidyapeeth (Deemed to be University), Pune, IND; 2 Otolaryngology-Head and Neck Surgery, Dr. D. Y. Patil Medical College, Hospital and Research Centre, Dr. D. Y. Patil Vidyapeeth (Deemed to be University), Pune, IND

**Keywords:** c.r. rao, diagnostic testing, epidemiology and biostatistics, padma bhushan, predictive model

## Abstract

This review article explores the seminal contributions of Professor C.R. Rao, one of the most influential statisticians of the 20th century, whose work has profoundly impacted statistical theory and its applications in various fields, particularly in medical statistics. This article discusses the theoretical foundations laid by Rao, including the Rao-Blackwell theorem, Cramer-Rao lower bound, and orthogonal arrays, and examines how these concepts have been applied to enhance medical research, from epidemiological studies to clinical trials. The article highlights the enduring relevance of Rao's work in improving the precision and efficiency of statistical methods used in medical research. The article also outlines his significant achievements and awards, underscoring his global statistical influence.

## Introduction and background

**Figure 1:**
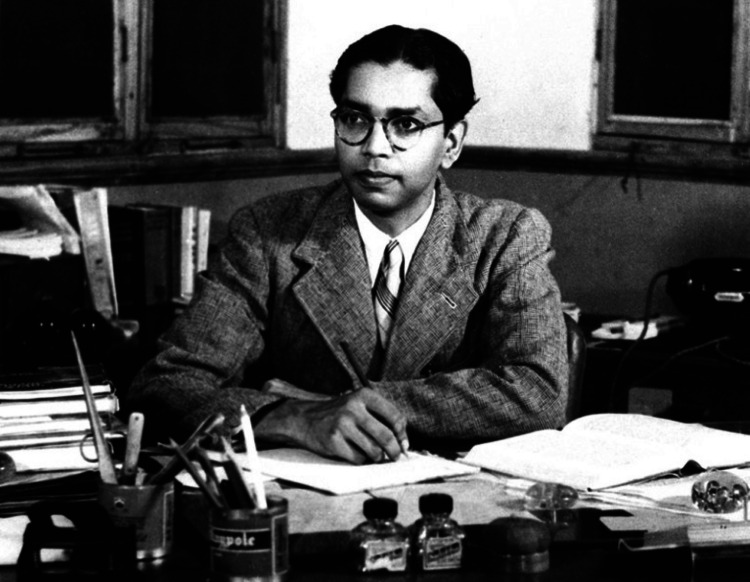
During his Indian Statistical Institute (ISI) days courtesy B.L.S.P. Rao Source: Reference [1]

Professor Calayampudi Radhakrishna Rao (Figure 1), often called C.R. Rao or Professor Rao, is one of the most distinguished and influential statisticians of the 20th century. His pioneering work in statistics has shaped modern statistical theory and practice [1]. His work has had far-reaching implications across multiple disciplines, including medical statistics, where precision and reliability are paramount [1]. This review delves into Professor Rao’s significant contributions to statistics. It illustrates how these contributions have been applied in medical research, enhancing the accuracy and efficiency of statistical analysis in this critical field [2].

## Review

Early life and academic career

Born on September 10, 1920, in Huvvina, Hadagali, then in the Madras Province, India, C.R. Rao’s early interest in Mathematics led him to have an MSc degree in Mathematics from Andhra University along with an MA in statistics from Calcutta University, and he was one of the first five students who got the Master’s degree from the University [1]. Professor Rao’s career in statistics progressed when he moved to Cambridge for a doctoral degree under the supervision of Professor Sir R.A. Fisher (Father of Modern Statistics). Under the mentorship of Professor P.C. Mahalanobis (Father of Indian Statistics), Rao developed some of the most influential theories that remain relevant today [3]. His early work laid the groundwork for a career spanning more than seven decades, during which he published more than 300 research papers and several books, making significant contributions to statistical theory and applications.

Major contributions to statistical theory

The Rao-Blackwell Theorem

The Rao-Blackwell theorem, developed in collaboration with David Blackwell, is a cornerstone of estimation theory that provides a method to improve an unbiased estimator by conditioning on a sufficient statistic [2]. This theorem has profound implications in various fields, including medical statistics, where reducing the variance of an estimator is crucial for reliable results [3]. The theorem is particularly valuable in medical research, where precise estimation of treatment effects or disease parameters is necessary for making informed clinical decisions [4].

The Cramer-Rao Lower Bound

Another of Rao's key contributions is the Cramer-Rao lower bound, which establishes a lower bound on the variance of estimators, providing a benchmark for the efficiency of statistical estimators [2]. This concept is crucial in medical statistics, which helps assess the quality of estimators used in clinical trials and epidemiological studies [1]. For example, when estimating the mean survival time of patients in a clinical trial, the Cramer-Rao lower bound can be used to determine the minimum variance that an unbiased estimator can achieve, ensuring that the statistical methods employed are as efficient as possible [5].

Orthogonal Arrays and Experimental Design

Rao’s work on orthogonal arrays has been instrumental in designing experiments, especially in medical research, where experimental designs must efficiently handle complex interactions among variables [6]. Orthogonal arrays are used to design experiments that minimize the number of trials needed to investigate the effects of several factors simultaneously, which is crucial in resource-constrained medical studies [3]. In clinical trials, for instance, orthogonal arrays can structure experiments to maximize the information obtained while minimizing the number of experimental units, leading to more effective and efficient studies [5].

Rao’s Score Test

Rao's Score Test, also known as the Lagrange Multiplier Test, is another significant contribution that allows statisticians to test hypotheses within a model without needing to fit the model under the alternative hypothesis [7]. This method is widely used in medical statistics to test the adequacy of models and ensure that the models used in research accurately represent the data [1]. For example, in evaluating the fit of a logistic regression model predicting disease risk, the Score Test can be used to test whether certain variables should be included in the model, improving the model's predictive accuracy [8].

Applications of Rao’s contributions in medical statistics

Clinical Trials

Clinical trials are a fundamental aspect of medical research, where the efficacy and safety of new treatments are tested [5]. Applying the Rao-Blackwell theorem in clinical trials helps improve the estimators' precision to measure treatment effects [2]. For example, in estimating the mean difference in treatment outcomes between two groups, the Rao-Blackwellized estimator would provide a more efficient and reliable estimate by reducing the variance [5]. This leads to more accurate conclusions about the treatment's efficacy, which is critical in approving new drugs and therapies [5].

Epidemiological Studies

Epidemiological studies were performed on related states or events in populations [3]. The Cramer-Rao lower bound is often used to assess the efficiency of estimators in epidemiological models, ensuring that the models used are as accurate as possible [2]. This is crucial in studies estimating disease prevalence or incidence, where precision is vital in drawing valid conclusions [4]. For example, in a study estimating the incidence rate of a rare disease, applying the Cramer-Rao lower bound ensures that the variance of the incidence rate estimator is minimized, leading to more precise estimates that can better inform public health interventions [4].

Diagnostic Test Evaluation

The accuracy of diagnostic tests is vital in medical practice, and statistical methods are used to evaluate these tests [1]. Rao's Score Test is frequently used to assess whether a diagnostic test model adequately fits the observed data, ensuring that tests are reliable and valid [7]. Additionally, the Rao-Blackwell theorem can be applied to improve the estimators of sensitivity and specificity, which are critical measures of a diagnostic test's performance [1]. For example, in evaluating a new diagnostic test for detecting cancer, using Rao-Blackwellized estimators can lead to more precise estimates of the test's sensitivity and specificity, ensuring that the test accurately identifies patients with and without the disease [1].

Meta-Analysis

A meta-analysis was performed on conclusions from combined evidence [9]. Using the Rao-Blackwell theorem in meta-analysis helps reduce the variance of combined effect estimates, leading to more robust and reliable results [2]. This is particularly important in oncology or cardiology, where meta-analyses pool data from various clinical trials to draw more generalizable conclusions about treatment effects [10]. For instance, when conducting a meta-analysis to assess the efficacy of a new drug in lowering blood pressure, applying the Rao-Blackwell theorem ensures that the combined estimate of the drug's effect is as precise as possible, leading to more reliable recommendations for clinical practice [10].

Predictive Modeling

Predictive models are increasingly used in medical research to forecast the risk of diseases based on patient characteristics [8]. Rao’s contributions, such as the Cramer-Rao lower bound, are essential in assessing the efficiency of these models, ensuring that predictions are as accurate as possible [2]. Improved estimators derived from the Rao-Blackwell theorem can also enhance the performance of these models, leading to better predictions of patient outcomes [8]. For example, in developing a predictive model for cardiovascular disease risk, applying the Cramer-Rao lower bound can help identify the most efficient estimators for the model parameters, leading to more accurate risk predictions that can guide patient management and treatment decisions [8].

Major achievements and awards

**Figure 2:**
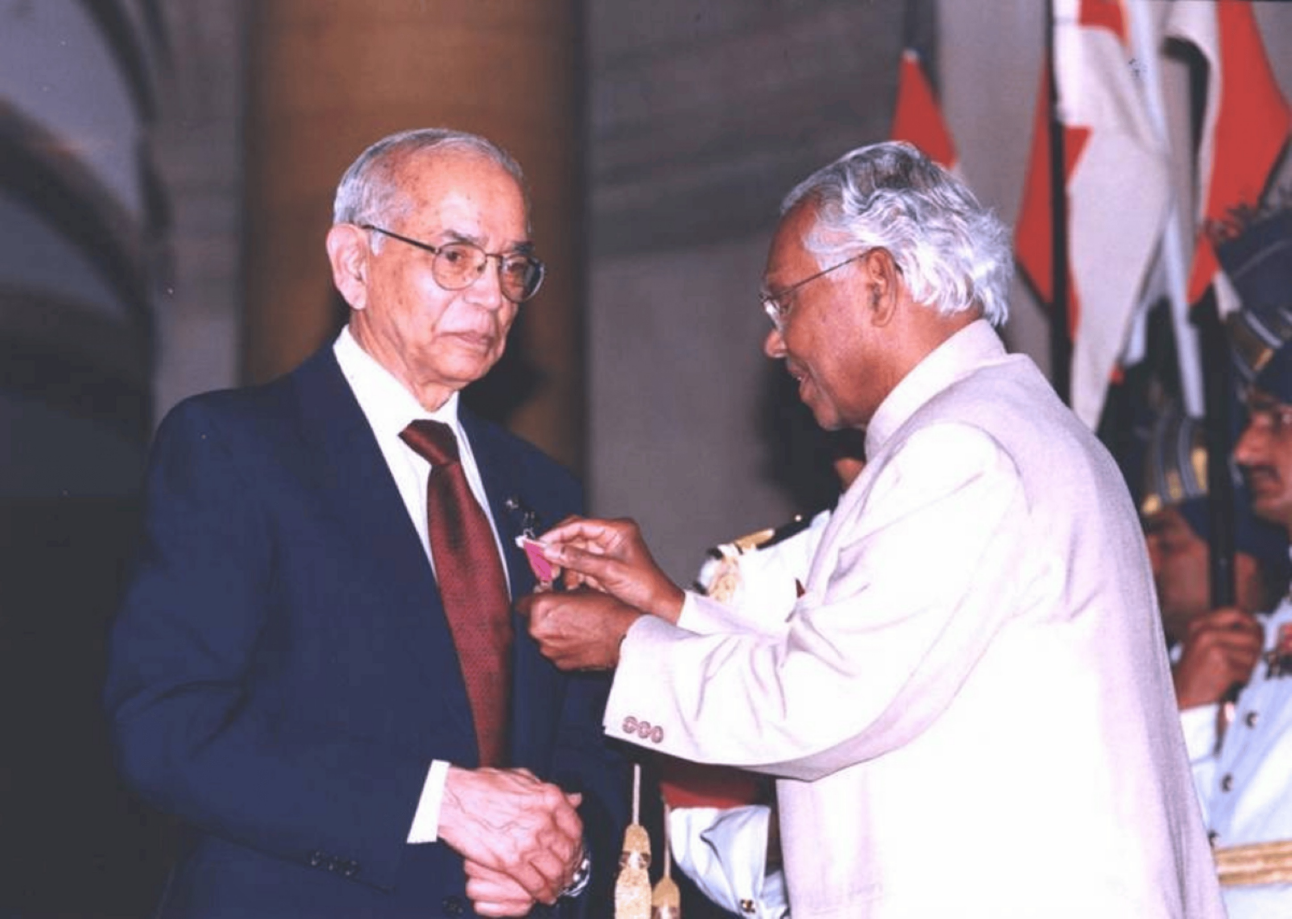
Professor C.R. Rao receiving Padma Vibhushan in 2001 from K.R. Narayanan. Courtesy B.L.S. Prakasa Rao Source: Reference [1]

C.R. Rao's illustrious career is marked by numerous prestigious awards and honors, reflecting his monumental contributions to statistics and their applications across various fields, including medicine. There were many achievements and awards, of which his most notable achievements were Padma Bhushan (1968) and Padma Vibhushan (2001) (Figure 2), two of India's highest civilian honors, recognizing Rao's exceptional contributions to science and education. The President of the United States awarded Rao the National Medal of Science (2002) to recognize and commemorate his pioneering work in statistics and its tremendous impact on different scientific fields, particularly medical research. The Royal Statistical Society bestowed the Guy Medal in Silver (1965) and Gold (2011) upon him for his excellent contributions to statistics. Fellow of the Royal Society (1967) elected Rao as a Fellow of the Royal Society, which is one of the highest honors a scientist can receive, reflecting his groundbreaking work in statistical theory. Rao was awarded the International Prize in Statistics (2023), also known as the "Nobel Prize of Statistics," for his significant contributions to the discipline, including the invention of the Cramer-Rao lower bound and Rao-Blackwell theorem.

These awards and honors are a testament to Rao's enduring influence in statistics and his contributions to medical research, where his work continues to enhance the quality and reliability of statistical methods.

The enduring legacy of C.R. Rao in medical statistics

The impact of C.R. Rao’s work on medical statistics cannot be overstated. His contributions continue to influence modern statistical practice, providing the tools necessary to improve the precision and reliability of medical research [1]. The methods he developed are now standard in the field, ensuring that medical studies are conducted with the highest level of statistical rigor.

As medical research continues to evolve, the foundational work of C.R. Rao remains a critical component of the statistical methods used to advance healthcare. Whether in the design of clinical trials, the evaluation of diagnostic tests, or the development of predictive models, Rao’s contributions provide the necessary statistical underpinnings to ensure that research findings are valid and reliable [4].

## Conclusions

Professor C.R. Rao's contributions to statistics have profoundly impacted the field, particularly in medical research, where his work continues to enhance the accuracy and efficiency of statistical methods. The application of his theories, such as the Rao-Blackwell theorem, Cramer-Rao lower bound, and Rao's Score Test, has significantly improved the quality of medical studies, leading to better healthcare outcomes. His numerous awards, including the Padma Vibhushan and the National Medal of Science, testify to his global statistical influence. As medical statistics continues to evolve, the principles established by Rao remain central to its development, ensuring that his legacy endures in the pursuit of statistical excellence in medical research. Professor Rao's life work exemplifies how theoretical statistical advancements can have profound practical applications, particularly in medicine, ultimately improving global healthcare outcomes.
